# Aggregation-Induced Emission Luminogens for Cell Death Research

**DOI:** 10.1021/acsbiomedchemau.1c00066

**Published:** 2022-02-24

**Authors:** Yunfei Zuo, Hanchen Shen, Feiyi Sun, Pei Li, Jianwei Sun, Ryan T. K. Kwok, Jacky W. Y. Lam, Ben Zhong Tang

**Affiliations:** † Department of Chemistry, Hong Kong Branch of Chinese National Engineering Research Center for Tissue Restoration and Reconstruction, and Guangdong-Hong Kong-Macau Joint Laboratory of Optoelectronic and Magnetic Functional Materials, Division of Life Science, and State Key Laboratory of Molecular Neuroscience, The Hong Kong University of Science & Technology, Clear Water Bay, Kowloon, Hong Kong 999077, P.R. China; ‡ Shenzhen Institute of Aggregate Science and Technology, School of Science and Engineering, The Chinese University of Hong Kong, Shenzhen, 2001 Longxiang Boulevard, Longgang District, Shenzhen City, Guangdong 518172, China; § Department of Gastrointestinal Surgery, The Second Clinical Medical College, Shenzhen People’s Hospital, Jinan University, Shenzhen, 518020, China

**Keywords:** aggregation-induced emission, cell death, fluorescence detection, bioimaging, therapy

## Abstract

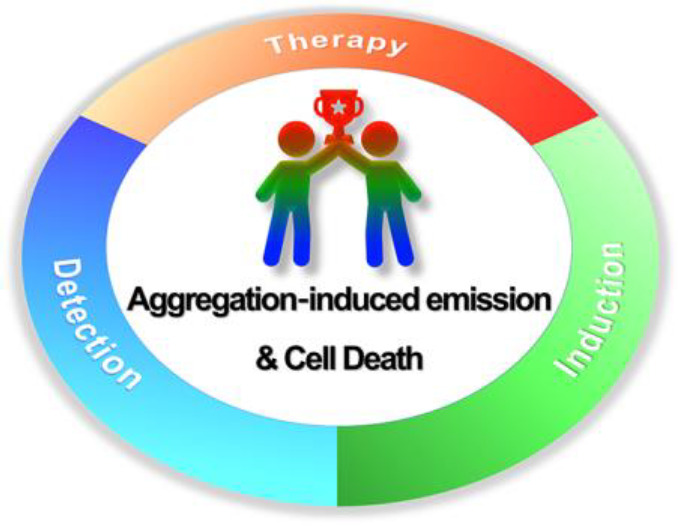

Cell death is closely related to various diseases, and monitoring and controlling cell death is a promising strategy to develop efficient therapy. Aggregation-induced emission luminogens (AIEgens) are ideal candidates for developing novel theranostic agents because of their intriguing properties in the aggregate state. The rational application of AIE materials in cell death-related research is still in its infancy but has shown great clinical potential. This review discussed the research frontier and our understanding of AIE materials in various subroutines of cell death, including apoptosis, necrosis, immunogenic cell death, pyroptosis, autophagy, lysosome-dependent cell death, and ferroptosis. We hope that the new insights can be offered to this growing field and attract more researchers to provide valuable contributions.

## Introduction

Cell death (CD) is an irreversible degeneration of vital cellular functions culminating in loss of cellular integrity (Table 1).^1−3^ According to traditional morphotype classification, cell death is classified into Types I, II, and III.^1,2^ Type I cell death is called apoptosis, and it exhibits distinguishing morphological characteristics including cell shrinkage, chromatin condensation, nuclear fragmentation, membrane blebbing, and apoptotic bodies.^2,4^ Type II cell death is also named autophagy-dependent cell death (ADCD) and manifested by large-scale cytoplasmic vacuolization to culminate in lysosomal degradation and cell death.^1,5^ Type III cell death corresponds to necrosis characterized by membrane integrity loss and subcellular organelle swelling.^2,6^ Depending on control mechanisms, cell death can be divided into accidental cell death (ACD) and regulated cell death (RCD).^1−3^ ACD is an instantaneous and biologically uncontrolled process corresponding to the plasma membrane breakdown caused by severe physical, chemical, or mechanical conditions.^2^ An example is represented by necrosis caused by infection or injury. In contrast, RCD relies on molecularly defined machinery that drugs or genetic interventions can modulate.^1,2^ RCD can be classified into various subroutines, namely, apoptosis, ADCD, necroptosis (regulated necrosis), pyroptosis, ferroptosis, immunogenic cell death (ICD), and lysosome-dependent cell death (LDCD).^3^

**Table 1:** Hallmarks of Different Types of Cell Death

cell death	key triggers	morphological features/hallmark events	ref
Apoptosis	Activation of caspases; Oxidative stress; DNA fragmentation; Phosphatidylserine exposure.	Cell shrinkage, chromatin condensation, nuclear fragmentation, membrane blebbing, and apoptotic bodies.	(2,3,39)
Necrosis	Oxidative stress; Calcium overload; Trauma or ischemia.	Swelling and rupture of the cell and its organelles, leakage of cellular contents, possibly triggering inflammation, no apparent lysosomal involvement	(2,3,6,7)
Pyroptosis	Activation of inflammasome; Activation of pro-inflammatory caspases; Oxidative stress.	Membranous pore formation, cytoplasmic swelling; rupture of the cell membrane and release of its intracellular contents into the immediate cellular milieu	(2,3,7,40)
Autophagy-Dependent Cell Death (ADCD)	Nutrient deprivation; Tat-Beclin **1**.	Lack of chromatin condensation, massive vacuolization of the cytoplasm, accumulation of autophagic vacuoles, little or no uptake by phagocytic cells	(2,3,41)
Ferroptosis	Oxidative stress and iron-dependent intracellular lipid peroxidation.	Loss of plasma membrane integrity, leakage of intracellular contents	(2,3,7,42)
Immunogenic Cell Death (ICD)	Viral infection; Chemotherapeutics (e.g., anthracyclines, bortezomib); Photosensitizers (e.g., temoporfin, chlorin e6, pheophorbide A, and hypericin).	Releases damage-associated molecular patterns to improve immunogenicity, translocation of calreticulin from endoplasmic reticulum to the surface of dying cells	(43,86,88)
Lysosome-Dependent Cell Death (LDCD)	Cathepsins; Oxidative stress.	Lysosomal membrane permeabilization, release of large lysosomal content into the cytosol.	(2,44,110)

Cell death plays a critical and fundamental role in regulating organism development,^7^ maintaining tissue homeostasis,^8^ eliminating potentially harmful cells,^9^ and controlling the aging process.^10^ Recently, cell death has attracted increasing attention in biological, pathological, and clinical studies because it is closely associated with various diseases.^1−3^ For example, selectively intercepting cell death by block caspase activation and caspase-dependent cell death (e.g., apoptosis and pyroptosis) can avoid neuronal death in the brain, which helps prevent Alzheimer’s disease.^11,12^ Increasing oxidative stress in the cell by generating reactive oxygen species (ROS) can selectively activate the cell death pathways of tumor cells, which can eliminate the malignant cells to provide effective cancer treatments.^8,13,14^ Thus, revealing the mechanism of cell death and disease and developing new treatments for controlling the cell death pathways promise humans prevention and therapy for as-yet-incurable diseases.

It is fundamental and vital to reveal cell death pathways and related physiological and pathological processes for developing applications in human disease therapy.^2,15−17^ Fluorescence techniques are primary bioimaging methods for real-time monitoring and revealing biological processes because of their high sensitivity, high resolution, noninvasive character, and on-site measurement.^18,19^ Organic fluorescent molecules are commonly used as probes due to their excellent synthetic diversity, outstanding biocompatibility, and large-scale commercialization.^18^ However, most traditional probes show weak or quenched emission in the aggregated state or at a highly concentrated solution because of the aggregation-caused quenching (ACQ) effect.^20−22^ For this reason, the ACQ effect limits the application of some organic probes based on conventional fluorophores like fluorescein and uranine. Thus, ideal probes that can precisely monitor biological processes and effectively treat disease are highly desired for better outcomes in disease treatments.^23−26^

**Scheme 1:**
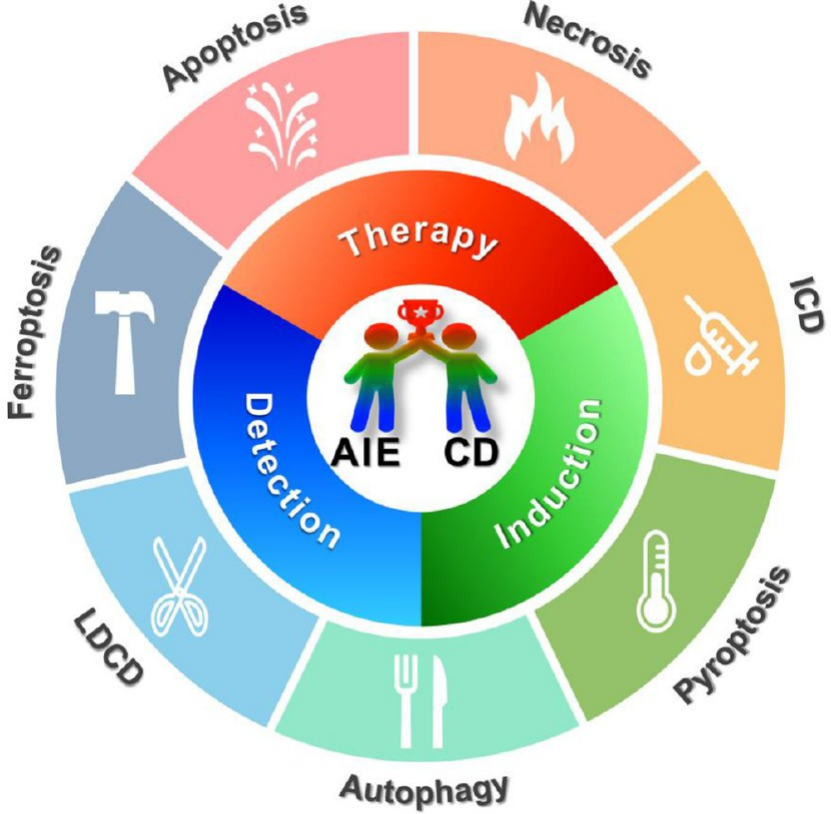
Applications of AIEgens in Major Cell Death Subroutines

In 2001, Tang found a photophysical phenomenon in which molecular aggregates exhibit stronger emission than single molecules, termed aggregation-induced emission (AIE).^20,27,28^ Unlike ACQ fluorophores, AIE luminogens (AIEgens) show bright emission in the aggregate state. Researchers have proposed a variety of explanations for AIE phenomenon in different organic systems. The restriction of intramolecular motions (RIM) is a widely accepted molecular mechanism.^20,27,29^ In this theory, the aggregation effectively suppresses intramolecular motions, which promotes the nonradiative decay of single molecules in the dilute solution. Recently, more detailed studies gave new insights into the AIE molecular mechanism, including restricted access to a conical intersection (RACI) and restriction of access to dark state (RADS).^30^ On the other hand, researchers began to notice that besides the single molecular structure, the molecular packing also largely influences the AIE phenomenon,^31^ showing more challenges and possibilities in designing AIE materials. During the last 20 years, AIEgens have garnered tremendous attention and achieved significant advances in biological applications.^21,22,32−35^ Compared to traditional ACQ fluorophores, AIEgens are ideal agents for fluorescence imaging and cancer theranostics because of their bright emission in the aggregate state, excellent photostability, large Stokes shift, high signal-to-noise ratio, and on-site activation ability.^18,23,24,36,37^ Additionally, many AIEgens are used as photosensitizers (PSs) to boost disease phototheranostics by generating high yields of cytotoxic reactive oxygen species (ROS).^23,38^ In a cell, excessive ROS can cause lipid peroxidation and damage to proteins and DNA to result in cell rupture finally.^2^ Furthermore, oxidative damage is not only a cause but also a result of multiform cell death.^2^ Compared with ACQ dyes, AIEgens can serve as ideal probes to monitor and trigger cell death processes. Since 2012, a growing number of novel AIEgens have been developed as indicators and inducers for various cell death subroutines, including apoptosis,^39^ necrosis,^6^ pyroptosis,^40^ autophagy,^41^ ferroptosis,^42^ ICD,^43^ and LDCD^44^ (Scheme 1, Table 1). Herein, we critically highlighted the recent examples of AIEgens for cell death researches and wished to arouse more people’s attention on this significant frontier.

## Apoptosis

Apoptosis was coined in 1972 by Kerr et al.^2^ It involves a complex signaling cascade amplification whereby extracellular or intracellular signals result in the orderly termination of cells.^45^ For multicellular organisms, apoptosis plays a significant role in controlling unwanted cell death during embryogenesis, growth, and tissue maintenance.^46^ Unregulated apoptosis may result in various pathologies and diseases.^45^ Insufficient apoptosis causes incorrect cell growth, fibrosis degeneration, and tumor formation, while excessive apoptosis may lead to degenerative diseases, such as degenerative disc and muscle atrophy.^45,47^ Overall, proper regulation of cell removal is essential, as both excessive and reduced apoptotic rates can lead to the onset of various diseases.

**Figure 1:**
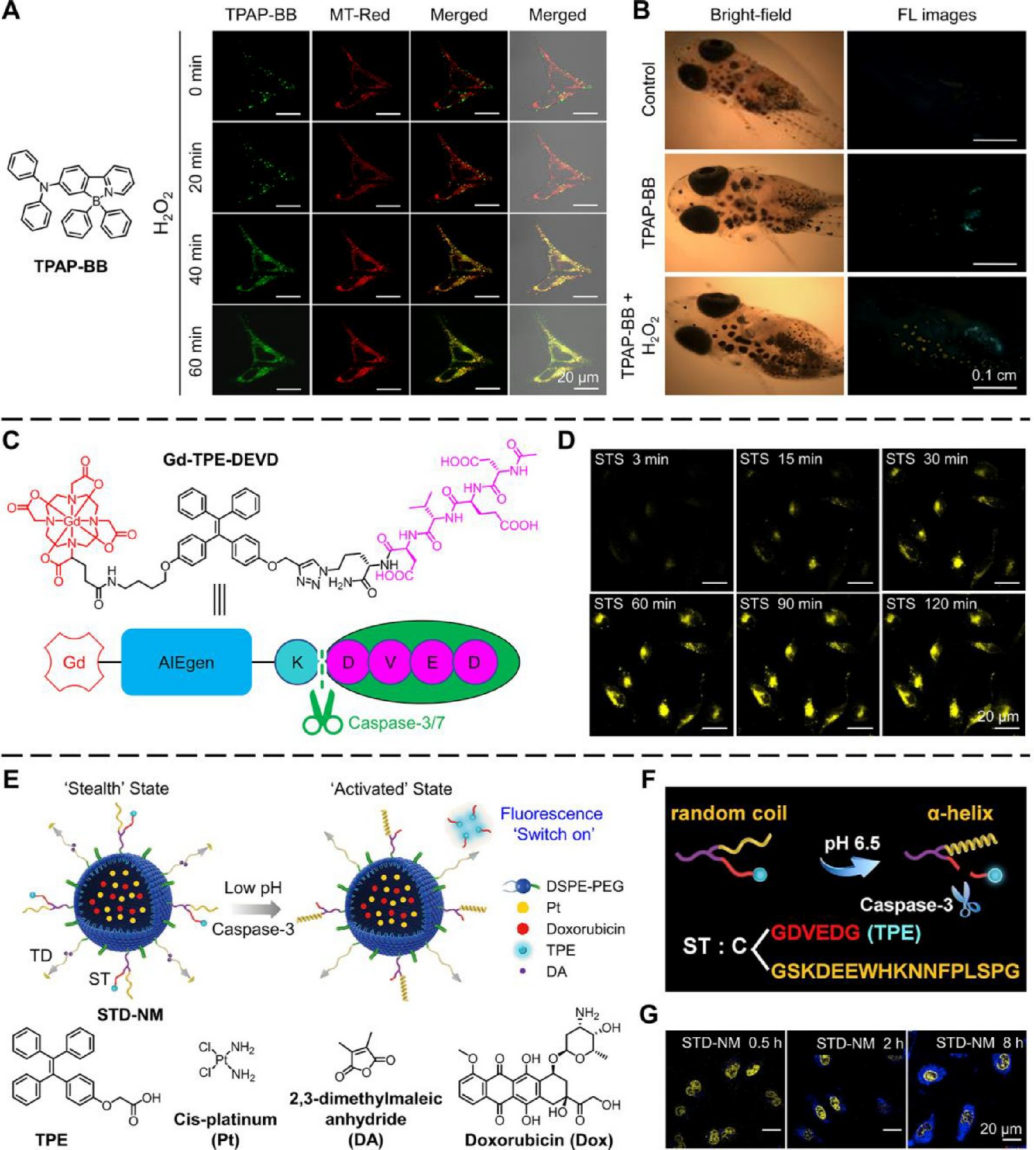
AIEgens for monitoring apoptosis. (A) Molecular structure of TPAP-BB and CLSM images of TPAP-BB- and MT-Red-stained HeLa cells displaying apoptotic progress. (B) Fluorescence microscopic images of TPAP-BB-stained Medaka fish without and with treatment with 2 mM H_2_O_2_ for 2 h. Adapted with permission from ref (48). Copyright 2018, American Chemical Society. (C) Molecular design of Gd-TPE-DEVD (CP1). (D) Fluorescence imaging of apoptotic HeLa cells incubated with CP1 (50 μM) before apoptosis induction with STS (1 μM). Adapted with permission from ref (53). Copyright 2019, American Chemical Society. (E) Scheme of tumor acidity/caspase-3 responsive STD-NM transition. (F) Peptide decoration format on the surface of STD-NM. (G) Real-time CLSM images of STD-NM stained HUVEC cells at pH 6.5 and nucleus (yellow) stained with DRAQ5. Adapted with permission from ref (55). Copyright 2019, Elsevier Ltd.

Real-time monitoring of the cell apoptosis is the first step to reveal the process. A wide variety of indicators have been designed based on different mechanisms.^48−60^ Cellular stress was found to trigger apoptosis and induce excess lipid droplets (LDs) in cells.^61^ Based on this understanding, in 2018, Dang et al. designed highly emissive LD-specific AIEgens called TPAP-BB (Figure 1A) for real-time specific detection of LDs and monitoring apoptosis induced at high H_2_O_2_ concentration (5 mM).^48^ Commercial MitoTracker-Red (MT-Red) was also employed to track the mitochondrial changes during apoptosis (Figure 1A). In the beginning, the emission of LDs (green color) and mitochondria (red color) was distinguishable. However, as apoptosis increased, the mitochondria showed green color from the TPAP-BB-stained LDs, suggesting LDs formation during apoptosis. Besides, “apoptotic bodies” were also successfully monitored by TPAP-BB during the apoptosis process. On account of the impressive monitoring of apoptosis *in vitro*, TPAP-BB was used to indicate apoptosis *in vivo*. As shown in Figure 1B, strong emission of TPAP-BB was observed in Medaka fish fed with H_2_O_2_-containing food, suggesting that the fish cells underwent apoptosis after treatment with H_2_O_2_.^48^ These findings proved that TPAP-BB achieved the real-time monitoring of apoptosis both *in vitro* and *in vivo*.

Caspases are a family of cysteine-dependent aspartate-specific proteases that play vital roles in initiating and executing apoptosis.^39^ Among them, caspase-3 and caspase-7 are essential executioners of various apoptosis.^2^ On the other hand, the Asp-Glu-Val-Asp (DEVD) peptide sequence is a hydrophilic caspase-3/7 responsive peptide linker. Theoretically, if a hydrophobic AIEgen is conjugated with DEVD, the probes will be water-soluble and nonfluorescent in an aqueous solution. Once the DEVD AIEgen unit is specifically cleaved by caspase-3/7, the hydrophobic AIEgen will emit strong light because of aggregate formation. Based on that strategy, many probes were developed for detecting the caspase-3/7 activities and monitoring apoptosis.^49,51,53,55^

In 2019, Meade et al. designed Gd-TPE-DEVD based on the strategy of turn-on response to caspase-3/7 for apoptosis imaging (Figure 1C).^53^ After cleaving the water-soluble peptide DEVD by caspase-3/7, the remaining hydrophobic Gd-TPE aggregated in aqueous solutions, which increased bimodal fluorescence-magnetic resonance signals. Furthermore, as shown in Figure 1D, the fluorescence intensity of HeLa cells gradually increased in a time-dependent manner when treated with the apoptosis inducer staurosporine (STS), demonstrating the success of detecting the activity of caspase-3/7 during apoptosis.

In addition to direct imaging, apoptosis imaging can be combined into complex systems, making a perfectly functional system. Wang et al. developed a smart nanomicelle called STD-NM with AIE property for cancer therapy and apoptosis imaging (Figure 1E).^55^ STD-NM encapsulated anticancer drugs of cis-platinum (Pt) and doxorubicin (Dox). On the surface of the nanocarrier are the peptides TD and ST. TD was acidity-activated cell-penetrating peptides comprising cell-penetrating peptides TAT and 2,3-dimethylmaleic anhydride (DA). ST comprised a pH-triggered targeting peptide STP (sequence: SKDEEWHKNNFPLSPG) and DEVD peptide sequence linked with an AIEgen of TPE (tetraphenylethylene) for “switch on” fluorescence in response to caspase-3 (Figure 1F), which could show “switch on” fluorescence during apoptosis. Once accumulated in the tumor acidic environment, peptide STP and TAT were activated, and the nanomicelle was in an “activated” state, which could enhance the cell permeability and further improve the penetrability of the “activated” STD-NM. As shown in Figure 1G, the real-time CLSM images of human umbilical vein endothelial cells (HUVEC) displayed caspase-3 specific recognition, and Pt drugs induced cellular apoptosis. So, the drug-encapsulated STD-NM showed a new design strategy for precise diagnosis and targeted therapy.

Except for monitoring apoptosis, AIEgens can also serve as apoptosis inducers based on two mechanisms.^4,62−65^ One is phototoxicity based on excessive cytotoxic ROS generated by AIEgens as photosensitizers (PSs) under light irradiation, and another is dark toxicity that can produce cytotoxicity without the participation of light.

**Figure 2:**
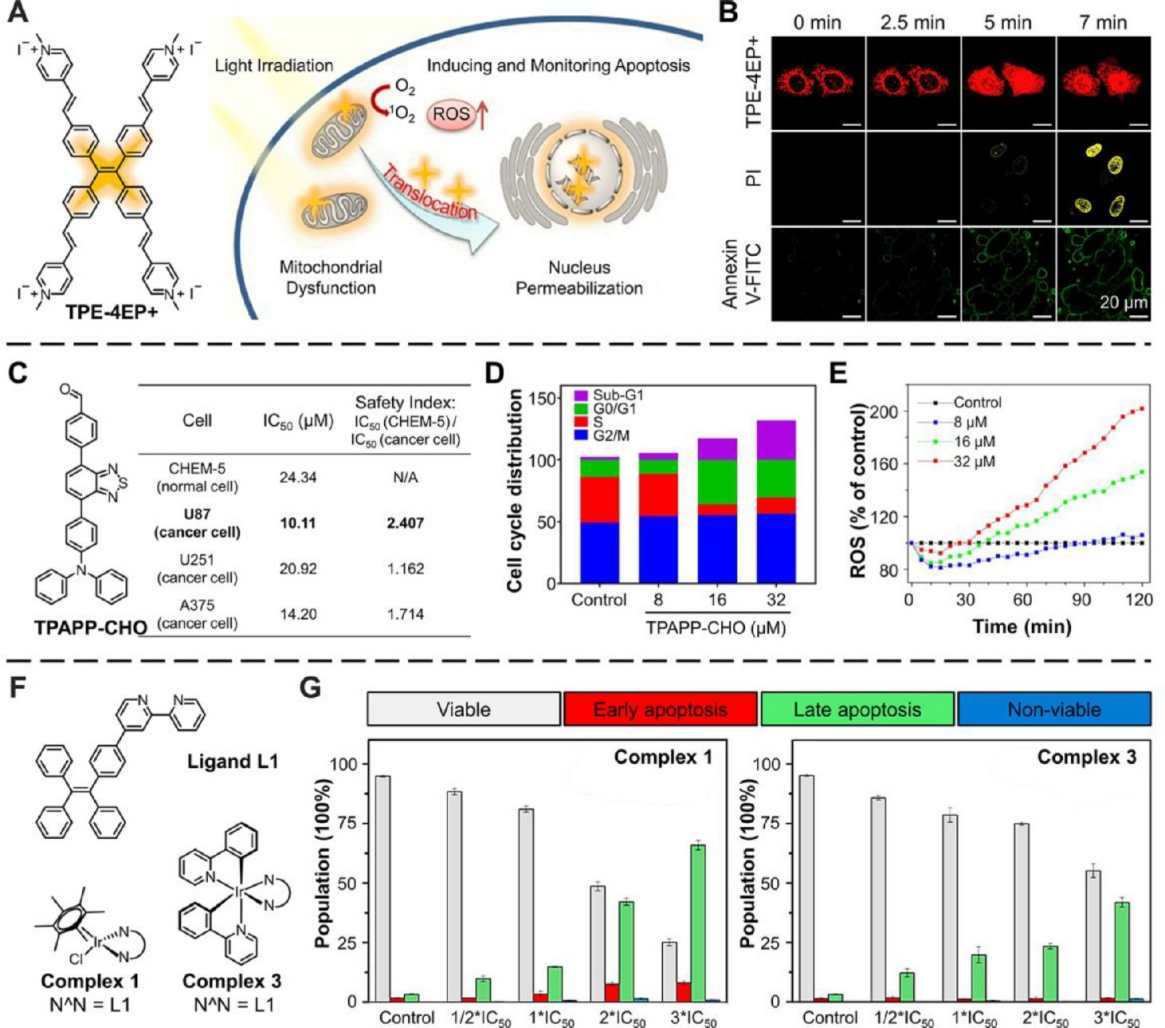
AIEgens for inducing apoptosis. (A) Molecular structures of TPE-4EP+ and mechanism of mitochondria-to-nucleus translocation. (B) Real-time CLSM images of HeLa Cells under continuous 405 laser irradiation stained with TPE-4EP+ (upper panel) and TPE-4EP+ followed by the addition of PI (middle panel) and Annexin V-FITC (ground panel). Adapted with permission from ref (4). Copyright 2019, American Chemical Society. (C) Structure of TPAP-CHO and cytotoxic effects of TPAPP-CHO NPs. (D) Flow cytometric analysis of apoptosis in U87 cells. (E) Activation of ROS overproduction by TPAPP-CHO NPS in U87 cells. Adapted with permission from ref (62). Copyright 2019, Wiley-VCH Verlag GmbH & Co. KGaA, Weinheim. (F) Structures of Ir(III) TPE complexes. (G) Flow cytometric analysis of A549 cells after 24 h of exposure to complexes 1 and 3 at 0.5, 1, 2, and 3*IC_50_ concentrations. Adapted with permission from ref (63). Copyright 2019, Wiley-VCH Verlag GmbH & Co. KGaA, Weinheim.

As shown in Figure 2A, TPE-4EP+ can induce apoptosis by generating a high amount of ^1^O_2_ under light irradiation.^4^ On the other hand, Annexin V can stain early-stage apoptotic cells, while PI only stains dead or late-stage apoptotic cells. Thus, Annexin V-FITC and PI were used to reveal the apoptosis process caused by the AIE probes under light condition (Figure 2B). First, TPE-4EP+ targeted mitochondria of the healthy cells. After 5 min irradiation, the annexin V-FITC signal appeared while the PI signal was still absent, indicating the early stage of the cell apoptosis. When lengthening the irradiation time to 7 min, an unmistakable PI signal appeared, and TPE-4EP+ was redistributed from mitochondria to the nucleus. This proved that the cells were in the late stage of apoptosis. Besides, flow cytometry was further carried out to confirm the cell imaging results. Briefly, TPE-4EP+ induced apoptosis and distinguished the different stages of apoptosis.

Another example of apoptosis was caused by the dark toxicity of AIEgens. An AIEgen called TPAPP-CHO formed nanoparticles (NPs) that could target lysosomes.^62^ In cytotoxicity assays, half-maximal inhibitory concentration (IC_50_) value is a quantitative measure that indicates the effectiveness of a compound in inhibiting biological or biochemical function. The IC_50_ in Figure 2C, demonstrated significant cytotoxicity of the NPs against human U87 cancer cells. Furthermore, the flow cytometric analysis showed that the population of sub-G1 phases was dose-dependent (Figure 2D). By increasing the concentration of TPAPP-CHO NPs, the sub-G1 phases accumulated, which indicated that apoptosis was the main reason for the anticancer effect. The ROS concertation in U87 cells not only increased with the treatment time, but also with the concentration of TPAPP-CHO NPs, suggesting that the NPs trigger intracellular ROS overproduction to result in cell apoptosis (Figure 2E).

In addition to organic AIEgens, metal-based AIE systems have recently attracted the interest of many researchers because of the potent anticancer activity of their organometallic unit.^63,66^ As shown in Figure 2F, Liu et al. designed four Ir(III) complexes from typical TPE derivatives and iridium(III) complexes.^63^ The flow cytometry results shown in Figure 2G demonstrated that Ir(III) TPE-containing complexes **1** and **3** induced a dose-dependent increase in the number of apoptotic cells. At a concentration of 3*IC_50_, complex **1** (IC_50_ = 3.56 ± 0.5 μM) induced 9.8% early apoptosis and 65.9% late apoptosis, while complex **3** (IC_50_ = 32.73 ± 0.5 μM) mainly induced 41.9% late apoptosis. This result was in agreement with the MTT assay, which further confirmed that these Ir(III) TPE complexes were apoptosis inducers and possible anticancer drugs.

**Figure 3:**
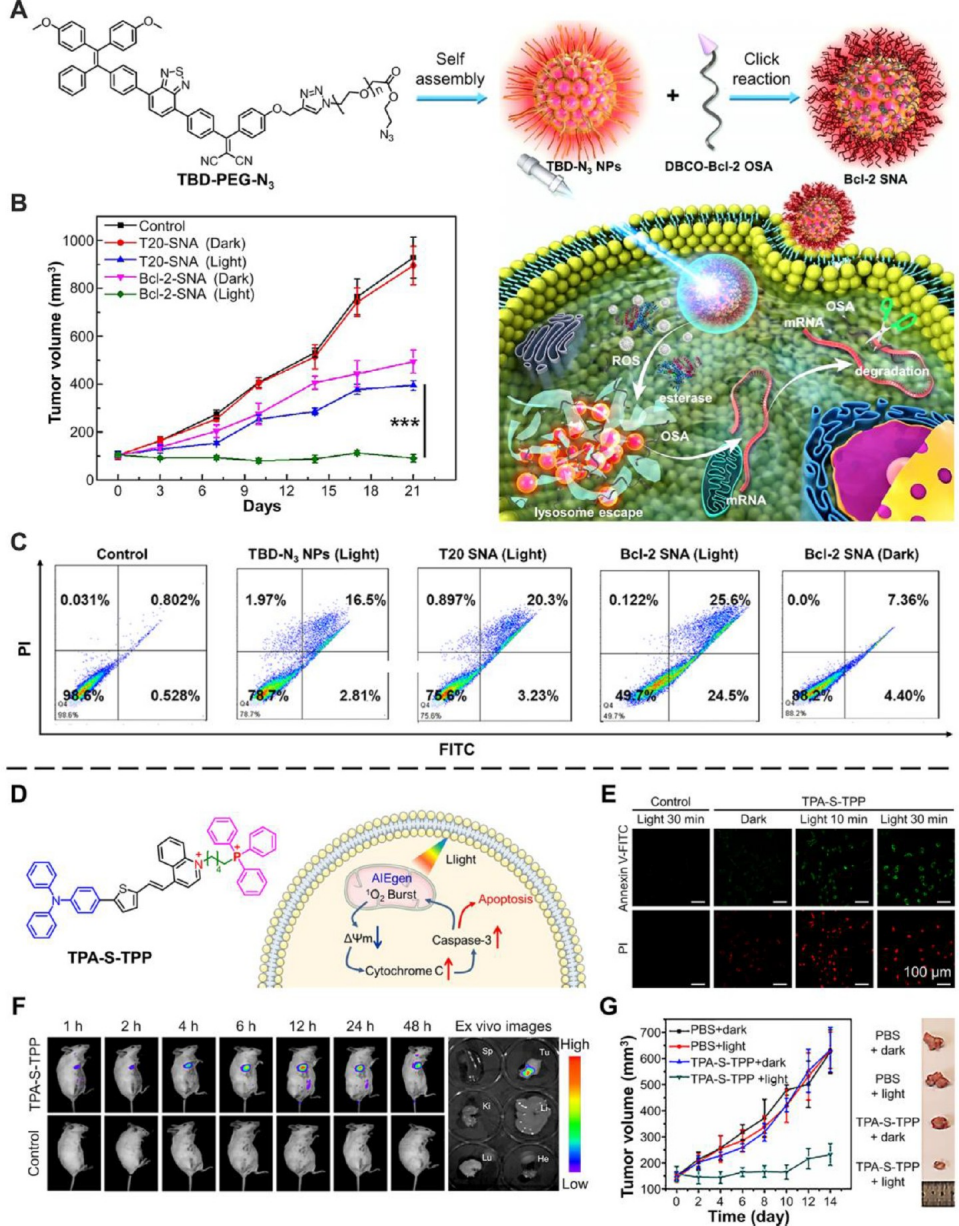
AIEgens for inducing tumor cell apoptosis for cancer therapy. (A) Structure of TBD-PEG-N_3_, preparation of AIE PS-based Bcl-2 SNA, and functional process of Bcl-2 SNA in tumor cells. (B) Tumor volume changes after injection of PBS, T20-SNA, Bcl-2 SNA with or without light illumination in tumor-bearing nude mice. The statistical significance level is ***p < 0.001. (C) Cell apoptosis study of HeLa cells after incubation under different culture conditions. Adapted with permission from ref (68). Copyright 2020, Wiley-VCH GmbH. (D) Structure of TPA-S-TPP and enhanced PDT in cells under light irradiation with AIEgen. (E) CLSM images of HeLa cells costained with annexin V-FITC and propidium iodide (PI) after different treatments. (F) Time-dependent *in vivo* images of 4T1 tumor-bearing BALB/c mice. *Ex vivo* images of the main organs (spleen: Sp; tumor: Tu; kidney: Ki; liver: Li; lung: Lu; heart: He). (G) Tumor volume changes of 4T1 tumor-bearing BALB/c mice during the different treatments (*n* = 5). Inset: Representative tumor photos of tumor-bearing mice after treatment for 14 days. Adapted with permission from ref (67). Copyright 2021, The Author(s). Published by the Royal Society of Chemistry.

Because AIEgens can monitor and induce apoptosis, they can treat diseases, especially in developing cancer therapy.^5,66−68^ As shown in Figure 3A, Bcl-2 antisense oligonucleotides (OSAs), which can decrease expression of the anti-apoptosis protein, were conjugated onto the surface of TBD-N_3_ NPs to form lysosome targeted SNA.^68^ The cell apoptosis assay showed that AIE NPs induced 21.3% apoptosis cell upon light illumination (Figure 3C). For Bcl-2-SNA, due to ROS production by AIE PSs, the lysosome ruptured, which promoted the escape of OSA, causing a higher apoptosis rate (50.3%). Thus, the AIE-based core–shell SNA could degrade the anti-apoptosis RNA and induce tumor cell apoptosis. Eventually, the tumor growth in the experimental group (Bcl-2-SNA) was almost completely inhibited, and the tumor inhibition rate was as high as 95% (Figure 3B), which proved the huge prospects for cancer treatment by controlling cell apoptosis.

For high-quality imaging in clinical use, NIR amphiphilic AIE photosensitizer named TPA-S-TPP with emission at 737 nm was designed to activate apoptosis, conduct *in vivo* imaging, and cure cancer (Figure 3D).^67^ Similar to Figure 2B, the same method was used to prove that TPA-S-TPP induced cell apoptosis upon light illumination (Figure 3E). Moreover, the decreased mitochondrial membrane potential and increased concentration of caspase 3 further proved that TPA-S-TPP was an apoptosis inducer.^67^ As illustrated in Figure 3F, compared with the control group, the NIR fluorescence signal of TPA-S-TPP at the tumor site gradually increased to confirm the potential of AIEgen for *in vivo* imaging and phototherapy applications. The photodynamic therapy (PDT) effect results showed that the tumor growth was significantly inhibited upon light treatment with TPA-S-TPP (Figure 3G), demonstrating a significant therapeutic effect. These results proved that AIEgens could therapy cancer by inducing tumor cell apoptosis.

As the primary type of RCD, apoptosis is widely used in disease treatments. However, due to some disease-related cells with inherent or induced resistance to apoptosis, the effects of apoptosis are sometimes limited, leading to unsatisfactory treatment results.^69,70^ Moreover, different diseases may have different single or mixed types of cell death.^2^ Therefore, revealing other cell death subroutines and developing novel therapeutics are essential for more effective disease therapy.

## Necrosis

In cancer therapy, evasion and resistance to apoptosis are almost inevitable.^71^ Because of this reason, research in cell death other than apoptosis is desired for efficient cancer therapy. Different from apoptosis, necrosis was traditionally thought of as an “accidental” type of death not regulated by molecular events.^71^ The feature of necrosis is cell organelle swelling, plasma membrane rupture, and eventually cell lysis. This process can also induce inflammation and tissue damage because of the spillage of intracellular contents into the surrounding tissue.^72^ Extreme physicochemical insults such as high-level ROS are highly possible to induce necrotic cell death. Thus, AIE-active photosensitizers (PSs) with strong ROS generation ability could help the development of new PDT agents. In this part, we reviewed AIE PSs with the ability to induce necrotic cell death. It should be noted that because the study of this kind of AIE PSs is still in its infancy and the exact cell death mechanism has not been well studied, the authors can only verify the rudimentary necrotic cell death. To better review these pioneering works, necrosis will be directly used with the same meaning of necrotic cell death in this review.

**Figure 4:**
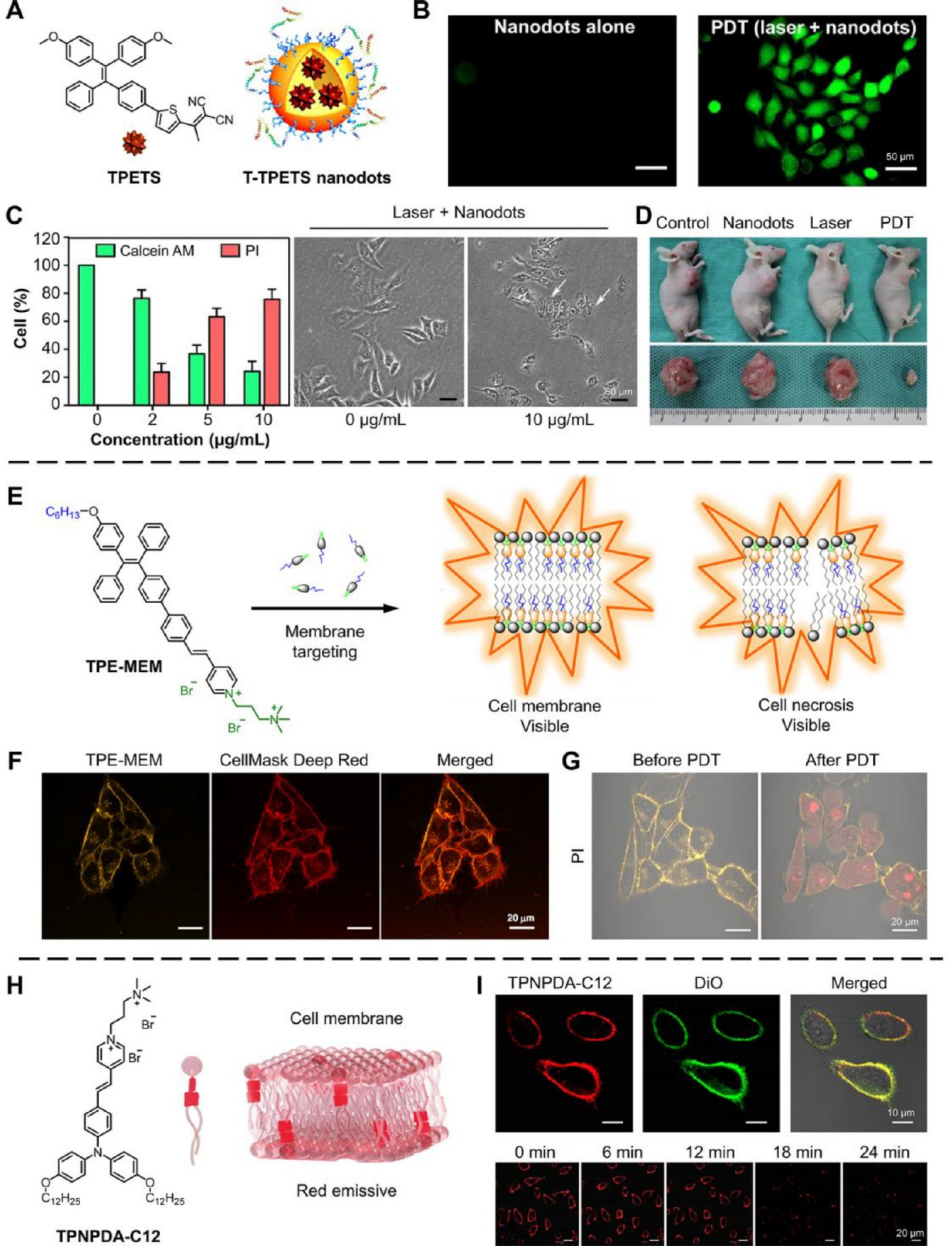
AIE-active organic small molecules for inducing and monitoring tumor cell necrosis for cancer therapy. (A) Structure of TPETS and schematic of T-TPETS nanodots. (B) Phototoxicity of T-TPETS nanodot *in vitro*. (C) Analysis of calcein-AM/PI staining in selected microscopic fields and TEM images after PDT treatment for 12 h. (D) Antitumor efficacy of T-TPETS nanodots *in vivo*. Adapted with permission from ref (73). Copyright 2019, Ivyspring International Publisher. (E) Schematic of the membrane targeting and necrosis monitoring effect of TPE-MEM. (F) Confocal images of HeLa cells using TPE-MEM (λ_ex_ = 405 nm and λ_em_ = 550 ± 70 nm) and CellMask Deep Red (λ_ex_ = 633 nm and λ_em_ = 685 ± 55 nm). (G) Confocal images of HeLa cells using TPE-MEM (λ_ex_ = 405 nm and λ_em_ = 550 ± 70 nm) and PI (λ_ex_ = 560 nm; λ_em_ = 620 ± 65 nm) before and after the PDT treatment. Adapted with permission from ref (74). Copyright 2019, American Chemical Society. (H) Structure of TPNPDA-C12 and schematic of the membrane staining effect. (I) Confocal images of HeLa cells using TPNPDA-C12 (λ_ex_ = 405 nm and λ_em_ = 490–560 nm) and DiO (λ_ex_ = 488 nm and λ_em_ = 500–600 nm), as well as the necrosis monitoring using TPNPDA-C12. Adapted with permission from ref (75). Copyright 2020, American Chemical Society.

Gao et al. encapsulated a strong AIE PS called TPETS into DSPE-PEG to form AIE nanodots and modify them with RGD motif (T-TPETS nanodots) for tumor targeting effect (Figure 4A).^73^*In vitro* test using 2′,7′-dichlorofluorescin diacetate showed that the prepared T-TPETS NDs could effectively induce ROS in cancerous HepG2 cells (Figure 4B). The authors utilized calcein-AM/propidium iodide (PI) staining in flow cytometry to test the cell viability and distinguish cells undergoing apoptosis and necrosis. The PI can only enter the cell with damaged membranes, which is a feature of necrosis cells. According to the flow cytometry results, the ratio of necrosis cells enhanced after increased the concentration of nanodot (Figure 4C). Further evidence of the cell morphological changes illustrated cell swelling in HepG2 cells with nanodots treatment, which is a hallmark of necrosis and keeps consistent with the flow cytometry results (Figure 4C). The necrosis induction also became stronger when lengthening the laser irradiation time, and all the results demonstrated that a higher ROS level could directly evoke cell necrosis rather than apoptosis. Tumor-bearing mice were used to confirm the *in vivo* antitumor effect of T-TPETS nanodots, and the results showed that the tumor growth was efficiently suppressed in mice by PDT treatment, while nanodots or laser irradiation alone could not inhibit tumor growth (Figure 4D). Since the loss of cell membrane integrity is highly related to necrosis, ROS generation in the cell membrane is thus highly possible to induce necrosis.

Zhang et al. designed and synthesized an amphiphilic AIEgen, namely, TPE-MEM, with the membrane monitoring and disrupting ability (Figure 4E).^74^ The finely tuned hydrophilicity rendered the AIEgen weakly emissive in water, and only after selectively binding with the cell membrane would the fluorescence of TPE-MEM be turned on. This property enabled high imaging quality of TPE-MEM as a cell membrane probe. In Figure 4F, the TPE-MEM clearly showed the membrane structure in HeLa cells, and the signal merged well with the signal from a commercial membrane-targeting dye called CellMask Deep Red. Due to the intrinsic ROS generation ability of TPE-MEM, the cell membrane was destroyed by the PDT treatment in the presence of normal white room light irradiation. As shown in Figure 4G, the TPE-MEM clearly showed the cell membrane structure before the PDT treatment, where PI could not enter the cell. However, after PDT treatment, the cell morphology obviously changed, and red emission of PI was observed because the integrity of the membrane was disrupted. In addition, the whole-cell showed red emission, which probably indicated the nucleus lysis into the cytoplasm. All these results proved that PDT induced cell necrosis under light irradiation.

Zheng et al. utilized a similar strategy to develop AIE PS to monitor cell necrosis.^75^ Besides yellow emission in mitochondria, the designed TPNPDA-C12 could show red emission when fused with the cell membrane (Figure 4H). The red fluorescence signals merged well with green cytoplasm membrane dye (DiO), and the signal could dynamically change with the integrity of the cell membrane (Figure 4I). The authors added 5 mM H_2_O_2_ in HeLa cells stained with TPNPDA-C12 to monitor the cell necrosis. It was found that the red fluorescence in the cell membrane gradually weakened and eventually disappeared over time, showing the fast cell necrosis process.

**Figure 5:**
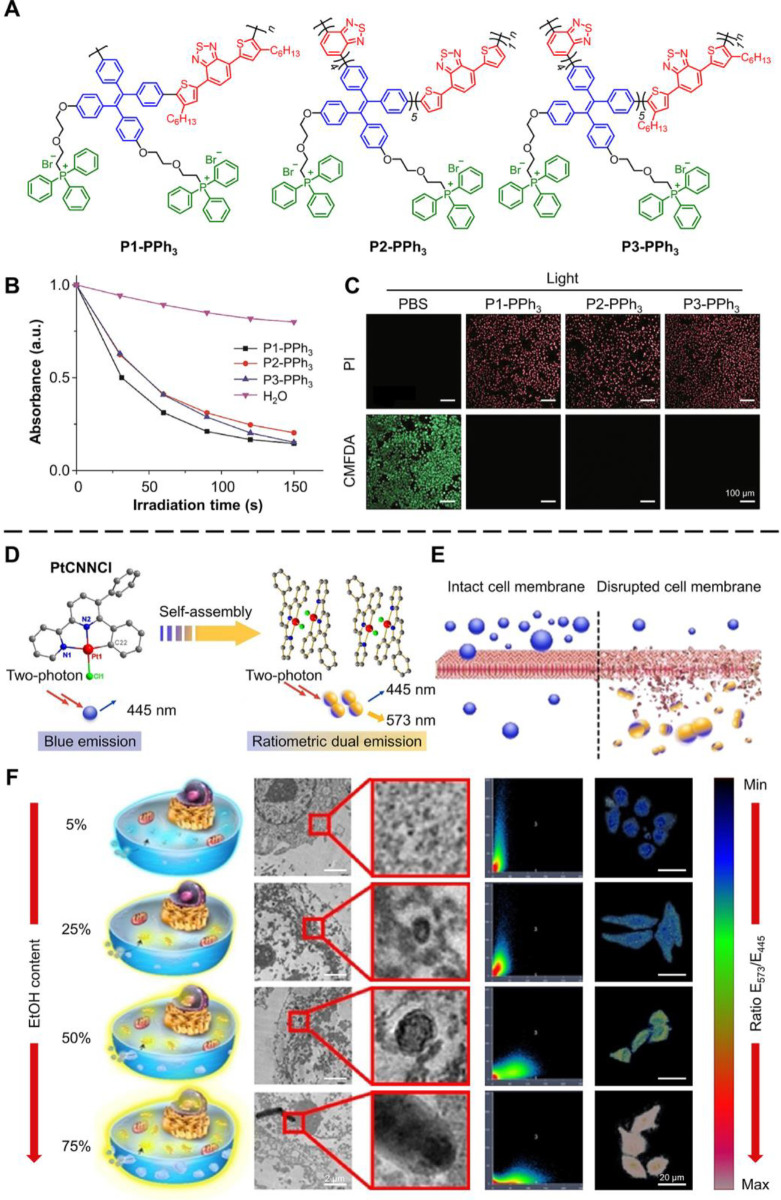
AIE-active polymer and metal complex for inducing and monitoring tumor cell necrosis for cancer therapy. (A) Structure of P1-PPh_3_, P2-PPh_3_, and P3-PPh_3_. (B) UV absorption peaks of ABDA after treatment with P1-PPh_3_, P2-PPh_3_, P3-PPh_3_, or H_2_O under white light irradiation. (C) Confocal images of HeLa cells stained by PI and CMFDA after treatment with PBS, P1-PPh_3_, P2-PPh_3_, and P3-PPh_3_ under white light irradiation. Adapted with permission from ref (76). Copyright 2020, Science China Press and Springer-Verlag GmbH Germany, part of Springer Nature. (D) Schematic of the two-photon excited ratiometric dual emission of PtCNNCl. (E) Schematic of necrosis cells recognization by PtCNNCl. (F) TEM images, scatter plot, and fluorescence intensity ratio (*E*_573_/*E*_445_) of HeLa cells, where *E*_573_ and *E*_445_ were the fluorescence intensity at the wavelength at 573 nm (yellow) and 445 nm (blue), respectively. Confocal images required two-photon excitation (λ_ex_ = 730 nm, λ_em_ = 445 ± 10 nm and λ_em_ = 57 ± 10 nm). Adapted with permission from ref (6). Copyright 2020, Wiley-VCH GmbH.

Besides small organic molecules, other AIEgens can also be used in cell necrosis studies. For example, Yao et al. developed three AIE conjugated polyelectrolytes (CPEs) for long-term tumor tracing and image-guided PDT (Figure 5A).^76^ These AIE-active CPEs boast superior water solubility, tumor retention effect, and high photostability, which are valuable for long-term tracing. On the other hand, CPEs always show enhanced fluorescence signal and ROS generation because of the fast energy transfer brought by the conjugated polymer backbone and large absorption coefficient. As shown in Figure 5B, all three AIE CPEs efficiently degraded the ROS indicator, namely, 9,10-anthracenediyl-bis(methylene)dimalonic acid (ABDA), under white light irradiation, which indicated their superior capability for ROS generation. Furthermore, the *in vitro* phototoxicity test showed that PI could stain the HeLa cells after PDT treatment using AIE CPEs (Figure 5C). This result indicated that the high level of ROS generated by AIE CPEs could kill cancer cells by inducing cell necrosis.

On the other hand, metal complexes play an essential role in the development of medicine, and many AIE-active metal complexes have shown great potential in biomedical applications.^77,78^ Ouyang et al. synthesized the organoplatinum(II) complex called PtCNNCl with blue emission and assembly induced yellow emission under one- or two-photon excitation (Figure 5D).^6^ At high concentration, PtCNNCl will form dimers and show yellow emission through the supramolecular assembly. PtCNNCl only emitted blue light when incubated in cells with intact membranes because of the saturation of cellular uptake (up to about 4 fg Pt/cell in HeLa cells). When the integrity of the cell membrane underwent disruption, more PtCNNCl could enter the cells and form yellow-emissive dimers inside the cells (Figure 5E). Thus, PtCNNCl can dynamically monitor the cell necrosis process. Various concentrations of ethanol (0%, 5%, 25%, 50%, and 75%) were used to treat HeLa cells to damage the membrane structure. The TEM images showed that the size of the PtCNNCl nanoaggregates was significantly increased at high ethanol concentration, and the fluorescence intensity ratio (*E*_573_/*E*_445_) was also increased (Figure 5F). Then, the authors treated the apoptotic and necrotic cells with PtCNNCl, respectively. Results found that the value of *E*_573_/*E*_445_ increased much faster in necrotic cells. Thus, PtCNNCl can be used to distinguish cell death pathways.

According to these results, the strong ROS generation and membrane targeting effect render the AIE PSs as ideal tools to induce cell necrosis, while the fluorescence signals sensitive to the external changes are helpful to distinguish and study cell necrosis.

## Immunogenic Cell Death

Chemotherapy using antitumor drugs is one of the most commonly used methods to treat cancers. The fundamental mechanism of many chemotherapeutic drugs is the activation of apoptosis.^79^ However, even with the exact mechanism, their therapeutic outcomes could be highly different because the tumor cells might gain drug resistance to apoptosis as we mentioned before. Even worse, many other therapies such as phototherapy, gene therapy, and radiotherapy also could introduce resistance to apoptosis.^69,80^ There has been sufficient clinical evidence to show that tumor-specific immune responses can largely determine the efficacy of anticancer therapies, and the special immune responses are highly related with immunogenic cell death (ICD).^81^ The immune system protects the human body from threats of the outside world like pathogens. The presence of pathogens can induce the immune system to clear invading pathogens and establish immunological memory for long-term protection.^82^ In the activation process, specific microorganism-associated molecular patterns (MAMPs) from the pathogens operating as natural adjuvants play important roles through the interaction with pattern recognition receptors (PRRs). The recognition by the immune system can arouse the first line of defense and make the initiation of antigen-specific immune responses possible.^83^ Similar to the pathogens, some cancer cells undergoing ICD can produce damage-associated molecular patterns (DAMPs), which can also be recognized by PRRs to initiate both innate and adaptive immune responses.^84^ Typical DAMPs include surface-exposed calreticulin (ecto-CRT), heat shock protein 70 (HSP70), adenosine triphosphate (ATP), and high mobility group protein B1 (HMGB1). As previously mentioned, the immune system can offer efficient and long-term protection. Consequently, the anticancer strategy based on ICD has demonstrated impressive treatment outcomes in patients suffering from highly aggressive cancer, like triple-negative breast cancer.^85^ Thus, induction of efficient ICD producing DAMPs provides a promising strategy for anticancer therapy. ICD inducers can trigger ICD mainly based on two mechanisms: (1) endoplasmic reticulum stress due to the formation of misfolded or unfolded proteins and (2) increased ROS during the therapy.^86,87^ As AIE PSs boast superior ROS generation ability and fluorescence signals for precise treatment, they can act as novel ICD inducers for cancer immunotherapy.^88^

**Figure 6:**
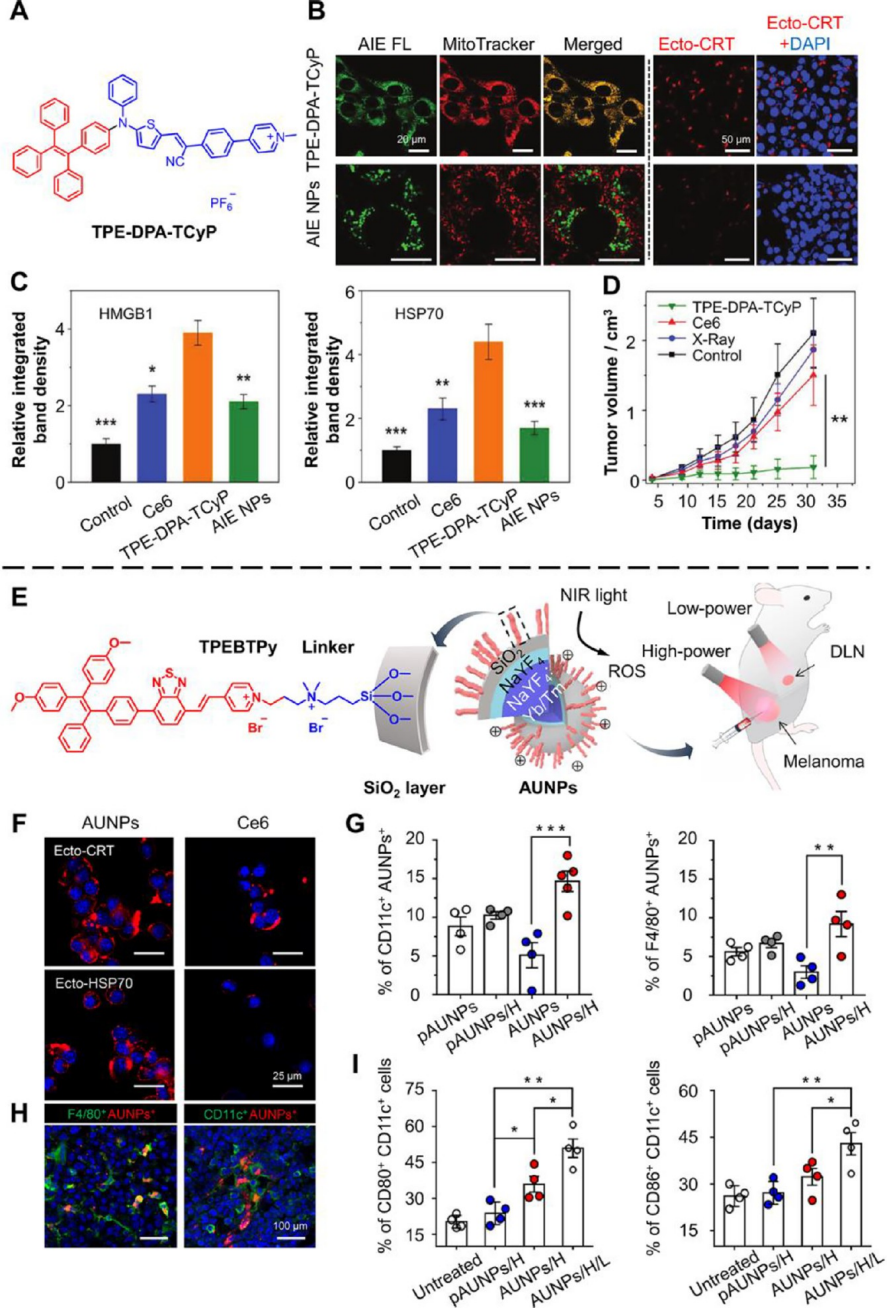
AIE-active materials for inducing tumor cell ICD for cancer therapy. (A) Structure of TPE-DPA-TCyP. (B) Confocal images of 4T1 cells treated with TPE-DPA-TCyP and AIE NPs (green), respectively, which were costained with MitoTracker Deep Red (red). Confocal images showing the ecto-CRT expression (red) were also shown. (C) Quantitative analyses of HMGB1 and HSP70 in the cell supernatants of 4T1 cells under different conditions. (D) Antitumor efficacy of TPE-DPA-TCyP *in vivo* using a prophylactic tumor vaccination model. (*P < 0.05, **P < 0.01, and ***P < 0.001.) Adapted with permission from ref (89). Copyright 2019, Wiley-VCH Verlag GmbH & Co. KGaA, Weinheim. (E) Schematic of the dual-mode ROS-driven tumor immunotherapy. (F) Confocal images of B16F10 cells treated with AUNPs under high-power NIR irradiation, showing the ecto-CRT expression and HSP70 (red). (G) Immunofluorescence images of DLN slices showing CD11c^+^ AUNPs^+^ DCs, and F4/80^+^ AUNPs^+^ macrophages. (H) FACS analysis of the percentages of CD11c^+^ DCs and F4/80^+^ macrophages containing AUNPs or pAUNPs in lymph nodes under different conditions. (I) FACS analysis of activated CD11c^+^ CD80^+^ DCs and CD11c^+^ CD86^+^ DCs in lymph nodes of B16F10 tumor-bearing mice with different treatments. (*P < 0.05, **P < 0.01, and ***P < 0.001.) Adapted with permission from ref (43). Copyright 2020, The Authors, some rights reserved; exclusive licensee American Association for the Advancement of Science.

Chen et al. designed and synthesized a mitochondria-targeting AIE PSs TPE-DPA-TCyP to study the influence of mitochondrial oxidative stress on ICD (Figure 6A).^89^ To ensure that the ROS generated in mitochondria can effectively induce ICD, the authors encapsulate the TPE-DPA-TCyP into F127 nanoparticles to prevent the AIE PSs from interacting with mitochondria (Figure 6B). Both TPE-DPA-TCyP and the corresponding AIE NPs could induce high ROS levels in cancer cells; however, the TPE-DPA-TCyP showed a much stronger ability to evoke ICD through the results of immunostaining analysis of ecto-CRT on the cancer cell surface (Figure 6B). The authors further studied other important DAMPs ATP, HMGB1, and HSP70. The results showed that all the DAMPs were significantly increased (Figure 6C). Furthermore, the TPE-DPA-TCyP exhibited much higher DAMPs induction than the commercial photosensitizer-based ICD inducer Ce6 and the AIE NPs, again showing the critical role of the synergetic effect of photosensitizing and mitochondria-targeting. Then the authors investigated the *in vivo* ICD immunogenicity of TPE-DPA-TCyP using a prophylactic tumor vaccination model. Only the TPE-DPA-TCyP exhibited a long-term antitumor immunity effect, efficiently suppressing tumor growth for 30 days (Figure 6D).

Mao et al. further explore the use of AIE-active ICD inducers in cancer immunotherapy.^43^ In their delicate design, the AIE PS TPEBTPy was linked to the SiO_2_ shell of the upconversion (UC) NPs through a quaternary ammonium linker to generate AIE luminogen (AIEgen)-coupled UCNPs (AUNPs). (Figure 6E). The UCNPs enabled the conversion of NIR light (usually 980 nm) to a shorter wavelength via the intermediate energy levels in lanthanide ions.^90^ The TPEBTPy could efficiently absorb the UC emission and generate corresponding ROS levels under high or low power 980 nm NIR irradiations for different therapy phases. The designed AUNPs realized photodynamic cancer immunotherapy using a NIR wavelength, which is valuable for treating large and deep-seated tumors. In addition, the positive charge on the surface of AUNPs was capable of capturing tumor-associated antigens (TAAs). Under high power NIR irradiation, the AUNPs evoked ICD in tumor cells, which was proven by upregulated DAMPs ecto-CRT and HSP70 (Figure 6F). AUNPs in this process would also capture the TAAs, and the AUNPs would then be taken up by antigen-presenting cells (APCs) to drain the lymph node (DLN) region. The immunofluorescence images of DLN demonstrated an enhanced AUNP uptake by dendritic cells (CD11c^+^) and macrophages (F4/80^+^) (Figure 6G). The *in vivo* fluorescence-activated cell sorting (FACS) test also confirmed this result, and the PEG-silane modified AUNP (pAUNP) could efficiently escape from APCs (Figure 6H). The role of positive charge on the NPs surface was thus shown. The following exposure to low-power NIR irradiation enabled the high expression of CD86 and CD80 on dendritic cells (DCs), indicating the successful *in vivo* DC activation by controlled NIR irradiation (Figure 6I). Further study has found that the AUNP could reduce immunosuppressive cells and relieve immune suppression. The combination with αPD-1 can enhance the antitumor effect to realize the long-term anticancer immunotherapy. The above data indicate that efficient induction of ICD played a central role in the successful application of this delicate immunotherapy system.

According to the reviewed works, evoking ICD can help construct a long-term antitumor effect. Because the ROS generated in a specific organelle plays a central role in regulating ICD, AIE PSs with strong ROS generation and stable fluorescence signal can act as ideal ICD inducers and provide researchers with more information about the ICD process.

## Pyroptosis

Pyroptosis is a form of caspase-1 dependent RCD driven by inflammasome activation with characterized morphologic change,^1,2^ and it has been reported to be closely associated with atherosclerosis and diabetic nephropathy diseases.^15^ Compared with apoptosis, pyroptosis could release cell contents and inflammatory cytokines, which trigger antitumor immune responses and help in cancer therapy.^40^ In a typical pyroptosis process, the active caspase-1 cleaves gasdermin-D (GSDMD) and releases N-terminal fragments (N-GSDMD) to form large bubbles at the plasma membrane (GSDMD pores), which can drive cell swelling, membrane ruptures, and eventually cell death.^2,40,91^ Recently, some studies found that some noncoding RNAs and other molecules in pyroptosis can promote pyroptosis and influence tumor proliferation, invasion, and metastasis.^15,92^ Thus, further research on pyroptosis will enhance our understanding of cancer and improve our treatment of cancers.

**Figure 7:**
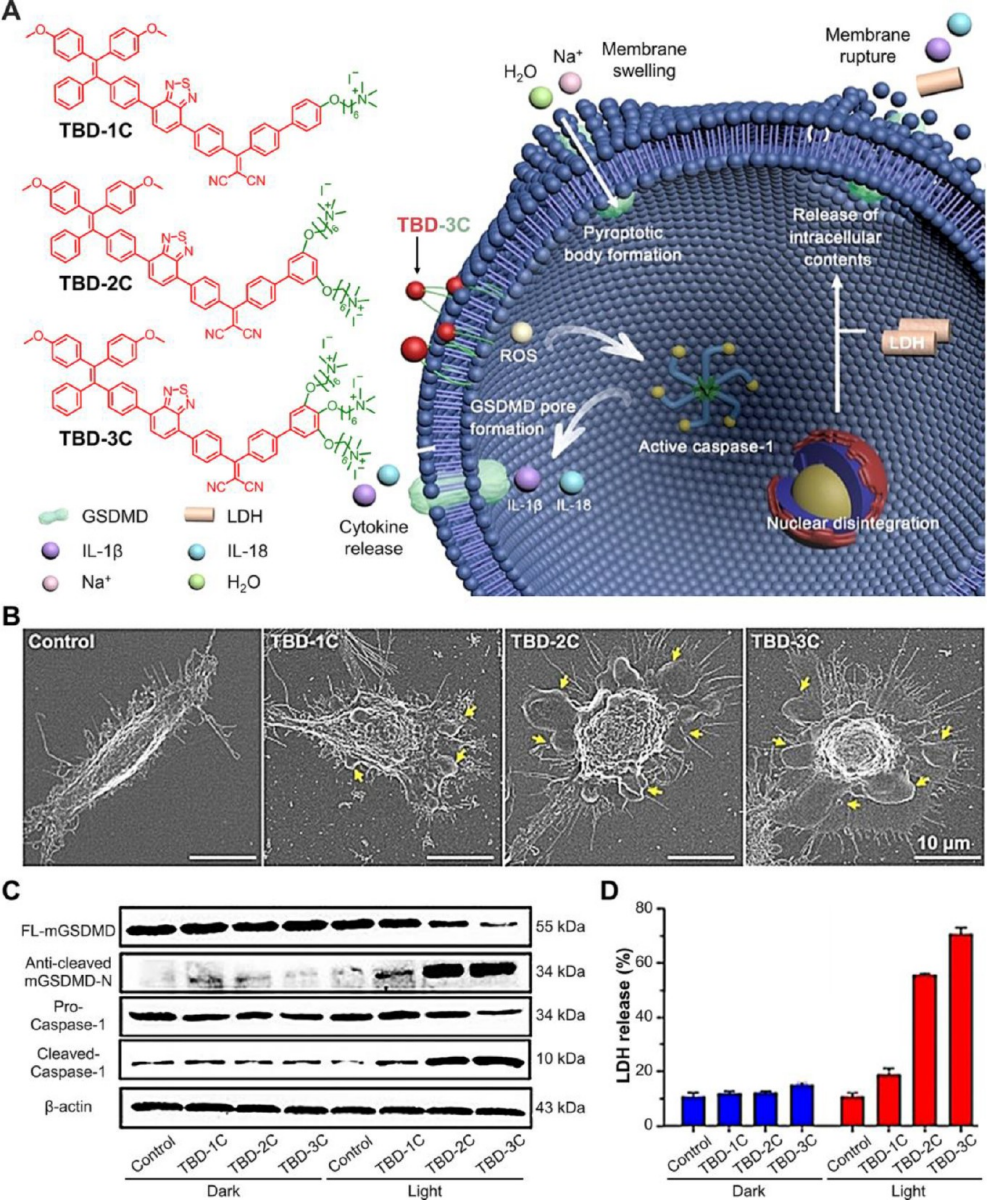
AIEgens for inducing pyroptosis. (A) Scheme of the membrane anchoring AIE photosensitizers activating pyroptosis. (B) Scanning electron microscopy image of HeLa cells with different treatments. (C) Cleavage of GSDMD and Caspase-1 were observed in response to PDT stimulation by Western blot assay. (D) Lactate dehydrogenase (LDH) release of HeLa cells after different treatments. Adapted with permission from ref (40). Copyright 2021, Wiley-VCH GmbH.

Zhang and Liu et al. reported an AIE probe that bioconjugated TPE and hydrophilic peptides for caspase-1 specific light-up.^91^ In 2021, Liu began to develop the application of AIEgens in the field of pyroptosis. As shown in Figure 7A, membrane-anchoring AIE PSs were designed to induce phospholipid peroxidation, damage the plasma membrane, trigger inflammasome activation, and eventually cause cell pyroptosis.^40^ In Figure 7B, upon treatment with TBD PSs under light irradiation, the cell morphology deformed and swelled with multiple bubble-like protrusions (pyroptotic bodies, indicated by yellow arrows) around the cell membrane. Compared to TBD-1C and TBD-2C, TBD-3C had enhanced membrane-anchoring ability because of more cationic chains. This property ensured TBD-3C to generate more ROS in situ to damage the cell membrane and promoting more obvious pyroptosis. Furthermore, Western blot assay results revealed that the expression of cleaved-GSDMD and cleaved-caspase-1 were significantly increased by enhancing PDT treatments (Figure 7C). Moreover, upon light irradiation, the level of lactate dehydrogenase (LDH, an indication of pyroptotic cell cytotoxicity) also remarkably increased, following the trend of Control < TBD-1C < TBD-2C < TBD-3C (Figure 7D). All those results confirmed that the membrane anchoring AIE PSs induces cancer cell death by activating the pyroptosis with oxidative stress.

We could see the great potential of activating pyroptosis for diseases therapy. Membrane targeting could be a sound molecular strategy to design AIEgens for pyroptosis research because the cell membrane damage could trigger the activation of the inflammasome, driving pyroptosis. On the other hand, caspase-1 is a critical player in pyroptosis and inflammation,^91^ which should be a good target for developing AIE indicators for pyroptosis. This largely neglected but important field needs more work.

## Autophagy

Autophagy is a conserved cellular catabolic process under various conditions, and it is highly related to multiple kinds of cell death pathways.^93^ This process delivers cytoplasmic material to the lysosome for degradation. The predominant function of autophagy is to promote cell survival under stress conditions. However, the accumulation of autophagosomes and autolysosomes in the cytoplasm of dying cells indicates a causal role for autophagy in the cell death process.^94^ According to numerous related studies, the relationship between autophagy and cell death can be mainly defined as three types:^94^ (i) autophagy-associated cell death, where autophagy happens together with other cell death pathways but does not have an active role; (ii) autophagy-mediated cell death, where autophagy would trigger apoptosis; (iii) autophagy-dependent cell death (ADCD), which is a type of RCD driven by the autophagic machinery. Thus, to prove the occurrence of ADCD, evidence that other cell death processes like apoptosis and necrosis are not involved or function in parallel is required.

Because of the complex functions of autophagy, researchers pay much attention to autophagy and try to develop new therapeutic methods for various diseases. For example, autophagy is an important process in cancer therapy. The exact roles of autophagy in tumors are complex and context-dependent. For example, Michaud et al. found that autophagy activation can trigger immunogenic cell death for efficient cancer therapy.^95^ On the other hand, sufficient results prove that the inhibition of autophagy or knockdown of autophagy genes would induce cancer cell death.^96^ Thus, the precise monitoring and even regulation of autophagy are valuable for modern medicine. AIE materials with superior fluorescence and various functions are ideal tools to achieve this goal.

**Figure 8:**
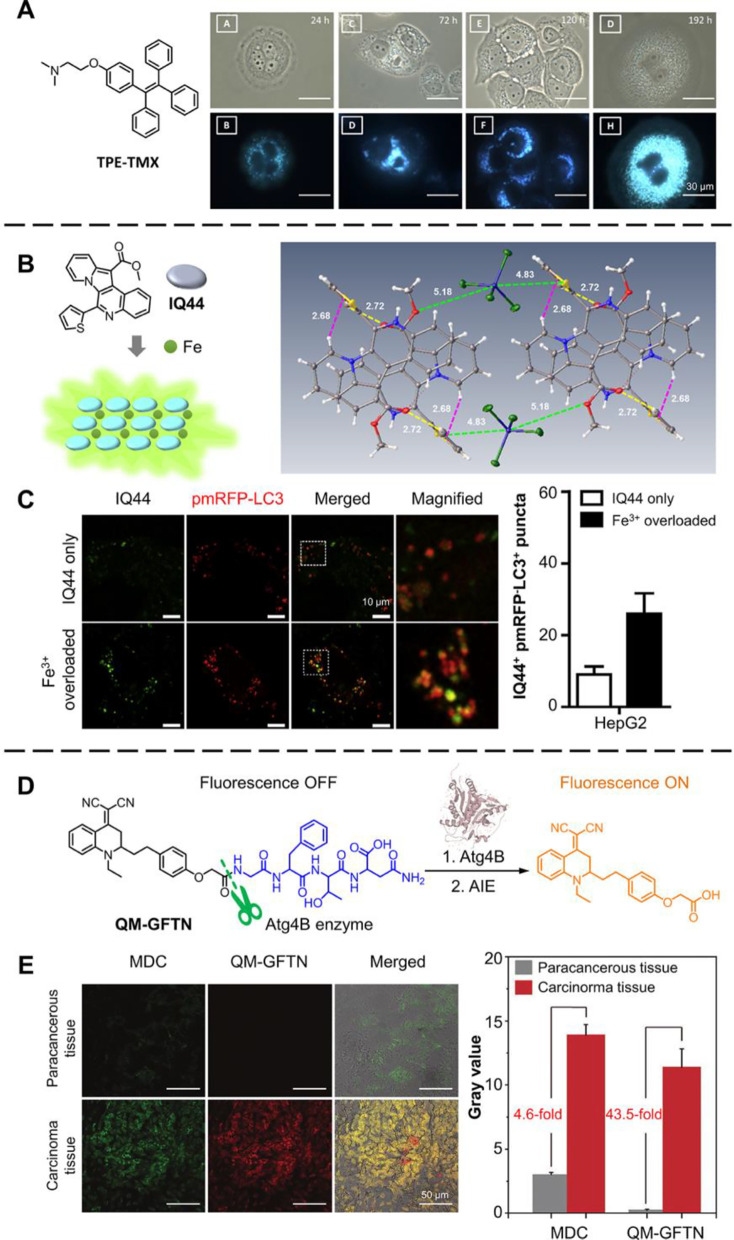
AIEgens for inducing and monitoring the autophagy process. (A) Structure of TPE-TMX, and fluorescence microscopy images of MCF-7 cells taken at different times treated by TPE-TMX (λ_ex_ = 330–385 nm, dichroic mirror: 400 nm and emission long-pass filter: 420 nm). Adapted with permission from ref (41). Copyright 2016, The Royal Society of Chemistry. (B) Structure of IQ44, the schematic of Fe^3+^ triggered turn-on process, and the X-ray structure of IQ44-Fe^3+^ complex. (C) Confocal images of HepG2 cells and Fe^3+^ overloaded HepG2 cells stained by IQ44 (λ_ex_ = 488 nm, λ_em_ = 493–560 nm) and pmRFP-LC3 (λ_ex_ = 561 nm, λ_em_ = 566–630 nm). Adapted with permission from ref (97). Copyright 2019, Elsevier Ltd. (D) Schematic of the detection of Atg4B using QM-GFTN. (E) Confocal images of tissues stained by MDC (λ_ex_ = 405 nm, λ_em_ = 450–550 nm) and QM-GFTN (λ_ex_ = 405 nm, λ_em_ = 650–750 nm), and the corresponding fluorescence intensity. Adapted with permission from ref (99). Copyright 2022, Wiley-VCH GmbH.

The mechanisms of some commonly used drugs are related to autophagy. For example, the chemotherapeutic agent Tamoxifen (TMX) was reported to induce autophagy in breast cancer cells. However, the exact process in treatment remains unclear because of the lack of reliable monitoring tools. To solve this problem, Zhao et al. designed and synthesized an AIE-active derivative of TMX called TPE-TMX (Figure 8A).^41^ The TPE-TMX showed the same lysosome targeting effect and cytotoxicity, and both of them showed cancer cell selectivity through the binding with estrogen receptor (ER) in MCF-7 cells. These results indicate a similar therapeutic mechanism of TMS and TPE-TMX. Tracking the fluorescence signal from the TPE-TMX thus offered promising insights into the exact mechanism of ER inhibitor. As shown in Figure 8A, swelling of lysosomes and formation of autolysosomes could be seen in long-term monitoring. In addition, almost all the MCF-7 cells were emissive after 192 h, while only weak emission could be found in the nuclei. The results seem to contradict the commonly accepted working mechanism in which it is necessary for the drug to enter the cell nucleus. This work not only realized dynamic monitoring of autophagy, but also offered new insights into ER inhibitors. Autophagy is also highly related to the endocytosis pathway, so this process is essential in studying the interaction between cells and surrounding substances. Iron ions boast important physiological functions, and iron overload and iron toxicity research are essential because of the relation to various diseases such as cancers and neuron degeneration. Lim et al. prepared an AIE turn-on fluorescent IQ44, which could be used for selective detection of cellular Fe^3+^.^97^ The interaction between IQ44 and Fe^3+^ could be studied using the X-ray structure shown in Figure 8B. The interaction indicated significant intermolecular interactions, indicating a strong RIM effect for the AIE phenomenon. It was found that when overloaded with Fe^3+^, the fluorescence signals from lit up IQ44 merged well with commercial dyes for specific staining of lysosomes. This result showed that most iron species go to the lysosomes. The authors further studied the colocalization of fluorescent protein-tagged LC3 proteins (pmRFP-LC3) with IQ44 (Figure 8C). LC3 (microtubule-associated protein 1 light chain 3) was required for elongation and maturation of the autophagosome, and would be conjugated with the membrane.^98^ It was found that when overloaded with Fe^3+^, the fluorescence of IQ44 was turned on and showed a higher overlap ratio compared with the experiment without Fe^3+^. The higher overlap ratio meant successful detection of the autolysosome and proved that autophagy was induced.

As mentioned above, LC3 is essential for the autophagy process. In fact, the functions of LC3 largely rely on successful cleavage by Atg4B cysteine protease, so visualizing Atg4B activity by fluorescence can help the real-time and highly specific investigation of autophagy. Based on this, Lyu et al. grafted hydrophilic Atg4B-triggered peptide Gly-Phe-Thr-Asn (GFTN) on the AIE fluorophore quinoline-malonitrile (QM).^99^ The prepared QM-GFTN dispersed well in an aqueous solution without showing fluorescence. However, when triggered by Agt4B, the QM core became hydrophobic so that the AIE fluorescence would be turned on due to the RIM mechanism (Figure 8D). QM-GFTN showed outstanding selectivity toward Atg4B, and obtained a much higher signal/noise (S/N) ratio than commercial autophagic vacuole dye dansylcadaverine (MDC). As shown in Figure 8E, the authors applied QM-GFTN to evaluate the autophagy level of paracancerous and carcinoma tissues in humans. Both MDC and QM-GFTN showed stronger fluorescence emission in carcinoma tissues (high autophagy level) than in para-cancerous tissues (low autophagy level), and the signals merged well. Compared with MDC, QM-GFTN exhibited a much higher S/N ratio, where the fluorescence intensity was 43.5-fold higher than that of para-cancerous tissue.

**Figure 9:**
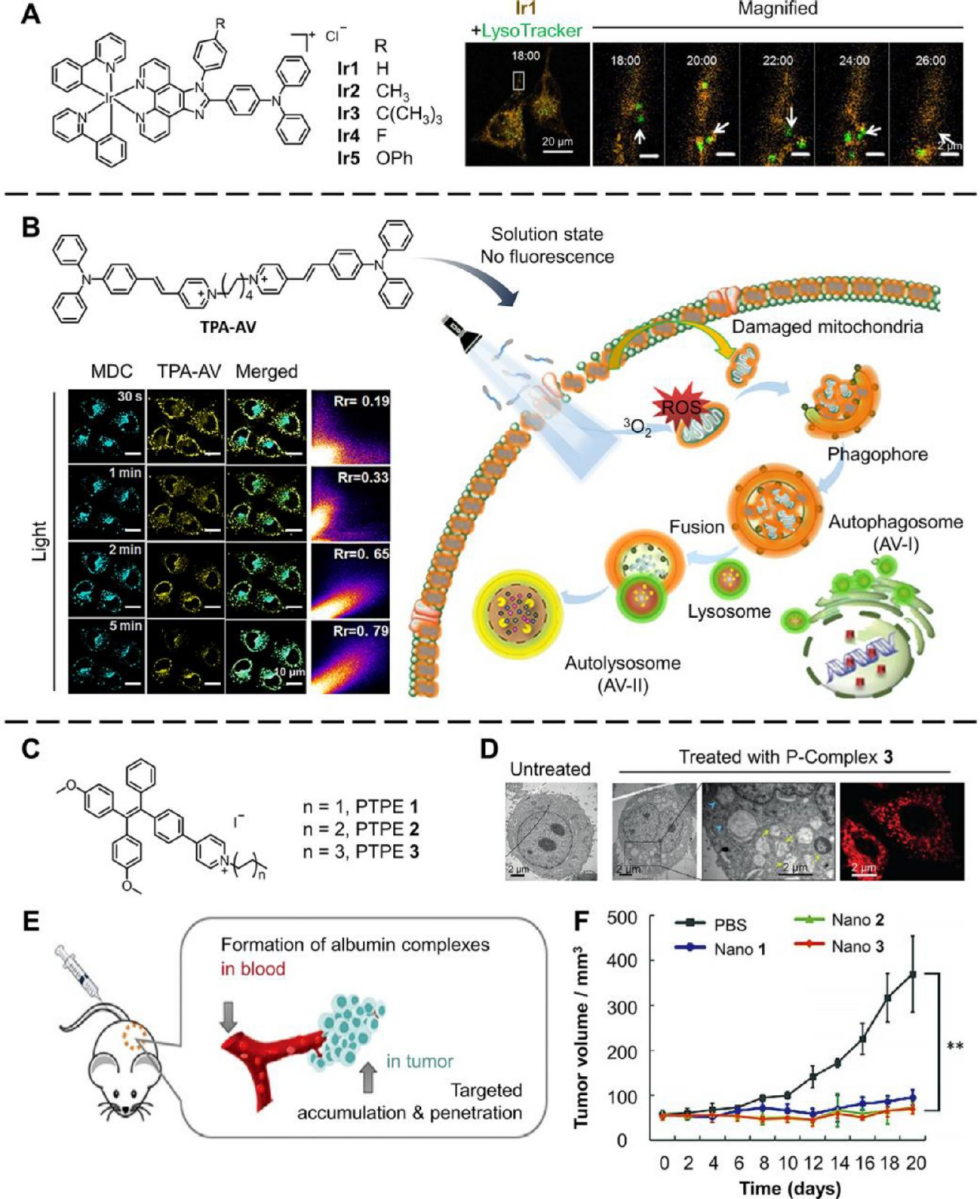
AIE materials for inducing and monitoring the mitophagy process. (A) Structure of Ir**1**–Ir**5**, and phosphorescence images of CCCP (10 μM) treated HeLa cells stained by Ir**1** (orange) and LysoTracker (green), respectively. Adapted with permission from ref (101). Copyright 2016, The Author(s). (B) Schematic of PDT and mitophagy monitoring by TPA-AV, and the confocal images of HeLa cells stained by MDC (λ_ex_ = 405 nm, λ_em_ = 500–530 nm) and TPA-AV (λ_ex_ = 488 nm, λ_em_ = 570–600 nm). Adapted with permission from ref (5). Copyright 2019, American Chemical Society. (C) Structures of PTPE **1**–**3**. (D) TEM images and confocal images of untreated HepG2 cells and HepG2 cells treated by P-complex **3** (λ_ex_ = 405 nm, λ_em_ = 550–600 nm). (E) Schematic of the complex formation and targeting antitumor therapy. (F) Antitumor efficacy of Nano **1**–**3***in vivo* (mean ± s.d., *n* = 4; **P < 0.01.) Adapted with permission from ref (102). Copyright 2020, Wiley-VCH Verlag GmbH & Co. KGaA, Weinheim.

Mitophagy is highly selective autophagy targeting damaged mitochondria, which is found to be downregulated in patients and models of Parkinson’s disease (PD).^100^ Many works have been done to monitor mitophagy using AIE probes. Due to the fact that some Ir(III) complexes boast a high mitochondria targeting effect and superior photostability, Jin et al. designed and synthesized a series of Ir(III) complexes as AIE phosphorescent probes to monitor the mitophagy process (Figure 9A).^101^ Ir**1**–Ir**5** all showed typical AIE effect, high photostability, and low cytotoxicity, and Ir**1** exhibited the best mitochondria targeting effect. These results mean that Ir**1** was suitable for bioimaging and monitoring mitophagy. To monitor the mitophagy triggered by carbonyl cyanide *m*-chlorophenylhydrazone (CCCP) in HeLa cells, Ir**1** and LysoTracker were used to locate the mitochondria and lysosome, respectively (Figure 9A). At about 18 min after the incubation, green LysoTracker-labeled lysosome (white arrow) overlapped with the orange Ir**1**-labeled mitochondria, which indicate the initiation of mitophagy and formation of the autophagosome. Most green fluorescence disappeared after approximately 26 min, suggesting the completion of mitophagy in this area of the cell.

Besides monitoring cell organelles, monitoring autophagy vacuoles (AVs), including autophagosome and autolysosome, is also valuable to study the mitophagy process. Wang et al. developed a series of membrane targeting AIE PSs to visualize AV formation in PDT treatment.^5^ TPA-AV showed the most suitable properties for biological applications and was used in the following experiments (Figure 9B). In the solution state, the TPA-AV showed no fluorescence because of the good dispersion, while the AIE fluorescence and ROS generation ability would be much enhanced when TPA-AV binds to the membrane structure. The TPA-AV would mainly stain the cell membrane without light irradiation. After 30 s of illumination, the commercial dye MDC indicated the appearance of AVs, and the localization of TPA-AV also started to change from the only staining cell membrane. Further, after 5 min of illumination, the high Pearson correlation factors value (*R*_r_ = 0.79) demonstrated that most TPA-AV stained the abundantly generated AVs. The following formation of autolysosome was also proved through the costaining using TPA-AV and LysoTracker, where the successful fusion of autophagosomes and lysosomes could be shown.

The mitophagy process can also be finely tuned to boost cancer therapy efficiency. Huang et al. synthesized three pyridinium-substituted tetraphenylethylene salts PTPE **1**–**3** as AIE PSs with different lengths of alkyl chains, which showed high mitochondrion affinity (Figure 9C).^102^ After binding with albumin, PTPE **1**–**3** could form corresponding P-complexes **1**–**3**, which exhibited tiny diameters smaller than 10 nm. Compared with untreated HepG2 cells, many autophagosomes (yellow arrows) and phagophores (blue arrows) could be seen in cells treated by PDT using P-complex **3** by TEM images (Figure 9D). This conclusion was also proved in confocal laser scanning microscopy (CLSM) images, where the mitochondria underwent a topological transformation into a vacuole-like structure. Interestingly, the P-complexes **1**–**3** were found to block the autophagy flux, where the fusion of autophagosome and lysosome would be inhibited. The autophagy flux process is important for cancer cells because suppression of the process can cause the accumulation of damaged organelles, which is highly harmful to cells. Directly adding PTPE **1**–**3** into PBS solution generated Nano **1**–**3**, respectively. As shown in Figure 9E, when entering the blood, the Nano **1**–**3** could form P-complexes **1**–**3** and target tumor tissues due to the enhanced accumulation and penetration. The *in vivo* murine tumor model showed that Nano **1**–**3** exhibited outstanding antitumor therapy effects (Figure 9F).

The superior fluorescence quality and photostability render AIEgens ideal for autophagy monitoring. These technologies help researchers to gain more insights into the physiological processes highly related to autophagy. Furthermore, some AIEgens can tune the autophagy process through certain structures, which can serve as pharmacophores, or through generating ROS. These properties can realize the future development of theranostic agents for various diseases, since autophagy is involved in many different diseases.

## Lysosome-Dependent Cell Death

Lysosome-dependent cell death (LDCD) is initiated by the release of hydrolytic enzymes (such as cathepsins) and iron into the cytosol after lysosomal membrane permeabilization (LMP).^1,2^ Importantly, LDCD introduced by LMP usually bypasses the classic caspase-dependent apoptosis pathway, providing a new promising anticancer strategy for overcoming apoptosis and drug resistant cancers.^3,103^ Many lysosome-targeted fluorescent AIEgens were used for tracking lysosomes,^104^ photodynamic therapy,^105^ and specific detection, such as subcellular organelle pH,^104^ endogenous HClO,^106^ heparin,^107^ β-*N*-acetylhexosaminidase,^108^ and carboxylesterases.^109^ However, LMP can also initiate or extend cell death processes, including apoptosis, ADCD, and ferroptosis.^2,105^ For example, when the permeability of the lysosome decreases, the toxic iron stored in the lysosome is released into the cytoplasm, resulting in ferroptosis.^2^ There were fewer research articles indicating LDCD probably because lysosome was related to many death processes,^110^ and different cell death might occur in “mixed” variants.^2^ It was also possible that other death modes dominated, which led to the obscure of the LDCD process.^111^

**Figure 10:**
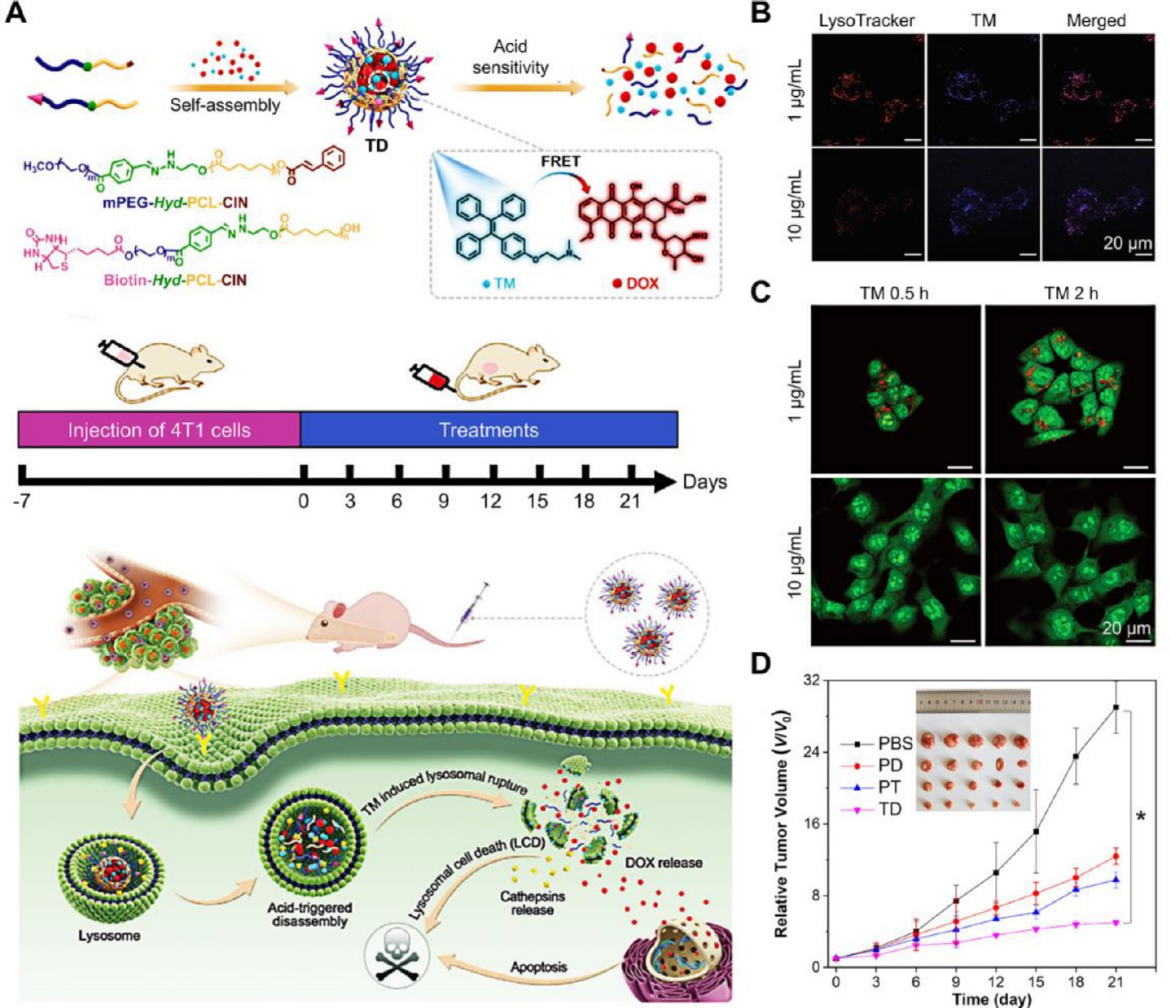
TM, an AIEgen, for lysosome-depended cell death. (A) Scheme of the preparation process of TD NPs, and its mechanism for anticancer therapy. (B) CLSM images of treated with LysoTracker Red for 0.5 h and then incubated with TM for 1 h. (C) 4T1 cells preincubated with TM followed by staining with acridine orange. (D) Time-dependent tumor growth curves. Insert: Representative tumor photos of tumor-bearing mice after treatment for 21 days. Adapted with permission from ref (44). Copyright 2021, Wiley-VCH GmbH.

In 2021, Li and Wu et al. explicitly presented an AIEgen that introduced LDCD for drug-resistant cancer therapy.^44^ As shown in Figure 10A, TM was an AIE-active and promising lysosome-targeting drug,^41^ and doxorubicin (DOX) was an anticancer drug distributed in the nucleus, inducing cell apoptosis. TD nanoparticles (NPs) were prepared through self-assembly by encapsulating TM and DOX with acid-responsive amphiphilic polymers. According to the 4T1 cell cytotoxicity test, at low concentrations (1 μg/mL), TM has negligible cytotoxicity while exhibiting remarkable cytotoxicity at high concentrations (10 μg/mL). When TM effectively targets the lysosome, the lysosome is damaged by 10 μg/mL TM, and the fluorescence of LysoTracker Red becomes weaker due to LMP (Figure 10B). TM could rupture the lysosome, which was further proven by staining with acridine orange (AO) that could penetrate acidic lysosome and emit red emission or show green fluorescence in the cytosol and nucleus. By increasing the concentation of TM to 10 μg/mL, the red signals almost disappeared, and only strong green emission confirmed that the lysosome integrity was destroyed, and LPM emerged (Figure 10C). So, with the help of LDCD introduced by TM and nuclear apoptosis caused by DOX, nanotheranostic system TD NP achieved excellent synergistic anticancer therapy effects (Figure 10D).

Other scientists also did lysosome-targeting for inhibiting tumor cell growth. For example, in 2021, Liu developed hybrid DNAzyme NPs that could rupture the lysosome structure and promote the NP escape from lysosomes that inhibited proliferation of cancer cells because AIE PSs produced ROS under light illumination.^112^ However, the cell death was apoptosis-induced by the PDT effect. Maybe there was an LDCD process, but more experiments are needed to prove this hypothesis. In the field where AIEgens introduced LDCD, further research is still required.

## Ferroptosis

Ferroptosis is an iron- and lipotoxicity-dependent non-apoptotic RCD.^2,3^ The possibility of cell ferroptosis depends on the balance between ROS production and the antioxidant system,^1^ and polyunsaturated fatty acids (PUFAs) are the prime targets of the lipid peroxidation of membranes. However, the molecular machinery of uncontrolled lipid peroxidation leading to ferroptosis is still in progress.^1,3^ Recently, ferroptosis has attracted overwhelming interest because it is related to various pathological conditions and diseases, such as ischemia/reperfusion injury (IRI), organ failure, neurodegeneration, and therapy-resistant tumors.^16,113^ More importantly, the ferroptosis pathway provides various druggable nodes for as-yet incurable diseases.^16^

**Figure 11:**
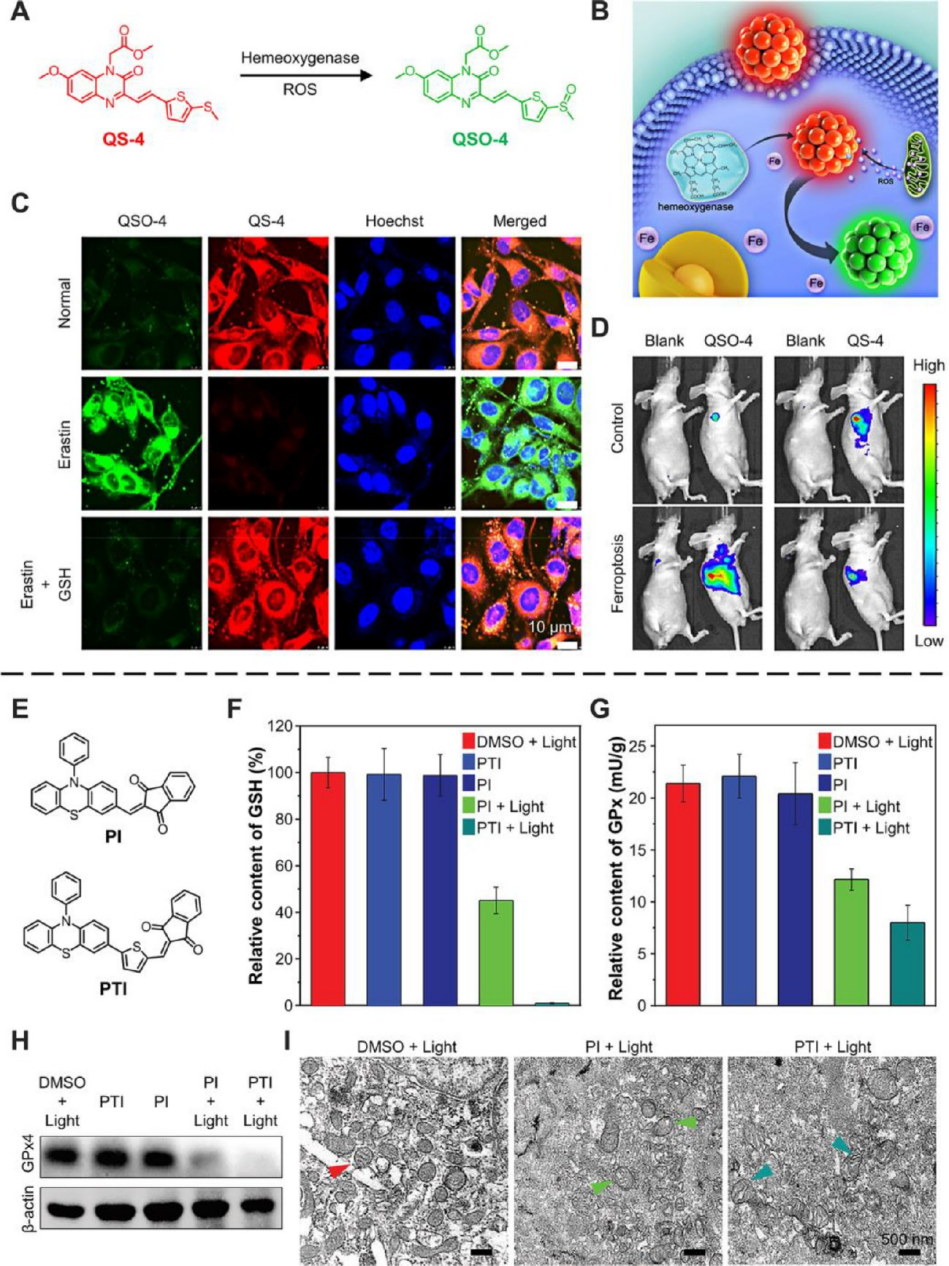
AIEgens for monitoring and inducing ferroptosis. (A) Transformation of QS-4 to QSQ-4. (B) Scheme of the transformation in ferroptosis cells. QS-4 for ferroptosis (C) *in vitro* and (D) *in vivo* imaging. Adapted with permission from ref (42). Copyright 2018, American Chemical Society. (E) Structure of PI and PTI. The relative content of (F) GSH and (G) GPx, and (I) TEM imaging of MCF-7 cells with different treatments. (H) Western blot detection of GPx4. Adapted with permission from ref (115). Copyright 2021, The Authors. Advanced Science published by Wiley-VCH GmbH.

AIEgens have already been used in some work in this field. For example, Zhang et al. designed a quinoxalinone-based fluorescent probe QS-4 for indicating ferroptosis (Figure 11A).^42^ QS-4 had a reactive aromatic thioether moiety, which could react with ROS and hemeoxygenase-1 (HO-1) and be oxidized into QSO-4, along with the nanodots’ emission color from red to green (Figure 11B). Erastin was commonly used to introduce ferroptosis by promoting the lipid peroxidation process, and GSH can eliminate peroxidation production. Therefore, when erastin induced the ferroptosis, the green fluorescence of QSO-4 increased significantly, and the red fluorescence was almost complete, indicating the cellular ferroptosis *in vitro* (Figure 11C). Moreover, the authors successfully applied the QS-4 nanodots to probe the ferroptosis *in vivo* (Figure 11D). According to the tumor slice confocal microscopy images, mice with ferroptosis exhibit enhanced green fluorescence and weak red fluorescence. All those great results show that QS-4 is a preferable AIEgen for monitoring cellular ferroptosis in living cells and animals. In 2021, Tong reported an AIE lipid order probe to detect cellular processes for real-time imaging cell death, including apoptosis and ferroptosis.^114^ They found that ferroptosis would not change cell membrane lipid transaction or striking morphology, different from apoptotic cells. The result might give some information for determining cell morphology in ferroptosis, as we still lack studies on distinguishing ferroptosis by morphological methods.^3^

Except for indicating ferroptosis, AIEgens could also be inducers. In 2021, Tang et al. reported two lipid droplet-targeting AIE PSs (PI and PTI) with excellent ROS generation (Figure 11E).^115^ They found PTI could induce cellular ferroptosis because high-level ROS oxidated LDs PUFAs and formed toxic lipid peroxides. Intracellular glutathione (GSH) and glutathione peroxidase (GPx) enzymes, especially GPx4, can protect biomembranes against peroxidation damage or increase the labile iron pool inactivated ferroptosis.^116^ Thus, the decrease of GSH and GPx indicated that the ferroptosis happened (Figure 11F–H). Moreover, mitochondrial morphology in the TEM images showed the abnormal mitochondria lacking cristae (cyan arrow) after PTI and light treatment (Figure 11I). Although the use of mitochondrial morphology to distinguish ferroptosis is still highly controversial because of the unclear correlation between mitochondria and ferroptosis,^3^ combining the expression of related proteins could prove the ferroptosis process. Besides, Liu found that a hybrid of ferric ions (Fe^3+^) and AIE PSs could probably enhance the PDT effect of AIE PSs due to the synergistic effect of ferroptosis.^117^

The research on ferroptosis is very hot because it is a significant unknown field. However, the usage of AIE for ferroptosis is relatively rare, and there are many places worth discovering and exploring.

## Conclusions and Perspectives

This review highlighted the recent examples of AIEgens used in studying the mechanisms and functions of cell death and aimed to attract more attention in this critical frontier field. Over the past decade, many AIEgens were used as indicators and inducers for cell death subroutines, including apoptosis, necrosis, ICD, pyroptosis, autophagy, LDCD, and ferroptosis. Most of them focus on anticancer therapy by inducing cell death and curing diseases of excessive cell proliferation. However, the regulation of cell death should be two-way. Inhibiting cell death to avoid cell loss is necessary for other significant disease type, but related research conducted by AIEgens is almost not found. The development of AIEgens as inhibitors of cell death will open another door. Additionally, the main research direction is related to apoptosis and anticancer therapy. However, other cell death pathways should also be studied since different diseases are contributed by different mechanisms, which always results from the loss of controllable single or mixed types of cell death. Therefore, we should expand design ideas and concepts and not confine them to such a narrow research field. In addition to the cell death described in the review, many other types of cell death, such as necroptosis, parthanatos, entosis, NETosis, alkali ptosis, and oxeiptosis, should also be further investigated. For example, the research on necroptosis is increasingly attractive because of the key role of necroptosis in various diseases. Necroptosis-based cancer therapy has been suggested as an alternative method to overcome apoptosis resistance, and trigger and amplify antitumor immunity in cancer therapy.^71^ On the other hand, necroptosis was found to contribute both directly and indirectly to neuronal loss, indicating the therapeutic potential for neuronal degeneration diseases.^12^ The development for AIE-active theranostic systems can help treat and understand these diseases. Last but not least, the major reported AIEgens were organelle targeting, while held for routine research but are not enough to study the molecular machinery of cell death, as the organelle change is not only a result but also a consequence of various types of cell death. Developing protein targeting AIEgens can reveal the role of excessive or deficient cell death in human disease. We believe that this review can provide an overall vision to the studies of AIE-active materials in the field of cell death, and we do hope that our perspectives can bring new inspirations to the researchers in this field. In summary, the authors hope that more scientists can provide contributions in developing AIE materials for cell death research.
